# A hybrid computational scheme for singularly perturbed Burgers’-Huxley equation

**DOI:** 10.1016/j.mex.2024.102574

**Published:** 2024-01-17

**Authors:** Imiru Takele Daba, Genanew Gofe Gonfa

**Affiliations:** Department of Mathematics, College of Natural Sciences, Salale University, Fitche, Ethiopia

**Keywords:** Nonlinear problem, Burger Huxley equation, Cubic-spline in tension method, Implicit Euler method, A Hybrid Computational Scheme for Singularly Perturbed Burger-Huxley Equation

## Abstract

This paper aims to construct and analyze a hybrid computational method for the nonlinear singularly perturbed Burgers’-Huxley equation. The presence of the perturbation parameter and non-linearity in the considered problem makes it difficult to solve the problem analytically and using classical numerical techniques on uniform step sizes as ε goes small. To elucidate such limitations, one can rely on non-classical numerical techniques. In this paper, one such parameter uniform numerical method is designed for the considered problem. The method is constructed:

• Firstly, the non-linear terms are linearized via Newton-Raphson-Kantorovich technique. The linearized problem is discretized by the implicit Euler method in the temporal direction.
• Secondly, the obtained equation is solved by employing the hybrid computational method comprised of the cubic spline in tension method in the inner layer region and the midpoint upwind method in the outer layer region on a piecewise uniform Shishkin mesh.
• Finally, an error analysis of the method is done and observed that the proposed method is parameter uniform convergent with the order of convergence $O\left(\tau +N^{-2}\ln^{3}N\right)$. Three examples are presented and the results are compared to some existing schemes in the literature to demonstrate the reliability of the proposed scheme.

Specifications table

**Table:** 

Subject area:	Mathematics and Statistics
More specific subject area:	Numerical Analysis
Method Name:	A Hybrid Computational Scheme for Singularly Perturbed Burger-Huxley Equation
Name and reference of original method:	Kaushik, A. and Sharma, M. (2008). A uniformly convergent numerical method on non-uniform mesh for singularly perturbed unsteady burger-huxley equation. Applied mathematics and computation, 195(2)688–706.
Resource availability:	MATLAB R2017a software

## Method details

There exists a class of singularly perturbed partial differential equations known as the nonlinear singularly perturbed Burgers’-Huxley equations (SPBHEs) that consist of nonlinearity and perturbation parameter, say ε(0<ε≪1) which is multiplied by the highest order derivative. These equations are an extension of the regular Burgers’ equation, which plays an important role in non-linear physics [13]. Due to their significance in a various scientific disciplines, the study of SPBHEs has got great practical interest among scientists. The non-linear SPBHE arises in many phenomena such as bursting oscillation [4], population genetics [1], interspike [18], bifurcation and chaos [23], membrane models based on the dynamics of potassium and sodium ion fluxes [16] and references therein. To investigate the behaviour of the physical phenomena of these problems, it is often vital to find their solution numerically. However, it is very tough to solve non-linear SPBHE analytically due to the presence of the perturbation parameter and the nonlinearity in the problem. As ε tends to zero, the classical computational techniques are fail to provide acceptable solutions for such types of problems. So, it is obligatory to develop robust numerical methods that solve the problems under consideration effectively.

In the literature, regular Burgers’ Huxley equations are well solved in numerous research works, some of which can be referred in [5], [7], [8], [9], [10], [14], [15], [19], [20], [21], [22], [24], [25], [26] for the detail discussions. However, very few parameter uniform numerical schemes are available for non-linear SPBHEs. For instance, Kaushik and Sharma [13] solved SPBHE using finite difference method (FDM). Gupta and Kadalbajoo [6] treated SPBHE by constructing hybrid parameter-uniform numerical schemes on a piecewise uniform Shishkin mesh. A uniformly convergent FDM on an adaptive nonuniform grid for Eq. (1) is presented by Liu et al. [17]. Jima and File, [11], Kabato and Duressa [12] and Derzie et al. [2] suggested a parameter uniform numerical schemes based on fitted operator techniques SPBHE. The use of numerical simulation for the analysis of dynamical systems of non-linear and non-smooth control problems has gained remarkable attention from scientific community. For further information, one can refer [28], [29], [30], [31], [32], [33].

In this work, we are motivated to develop and analyse an ε−uniformly convergent numerical scheme for SPBHE. Developing parameter uniform numerical techniques for SPBHE is a desirable task and active for research. A detailed description of the formulation of the scheme and analysis of the scheme is given in the subsequent sections.

**Notations** Throughout the paper, ${∥\cdot ∥}_{\infty }$ is defined by ${∥\mu ∥}_{\infty }=\sup\left(u,v\right)\left|\mu (u,v)\right|$ denotes standard supremum norm for a function μ defined on some domain η. N and M denote the number of mesh points in space (u) and time (v) directions, respectively. c denotes a generic positive constant independent of the ε and the mesh sizes.

### Continuous problem

In this paper, we examine SPBHE of the form

(1) $$\left\{ \begin{array}{c} {£}_{\varepsilon }z\left(u,v\right)\equiv \frac{\partial z}{\partial v}\left(u,v\right)-\varepsilon \frac{\partial^{2}z}{\partial u^{2}}\left(u,v\right)+\alpha z\frac{\partial z}{\partial u}\left(u,v\right)-\theta \left(1-z(u,v)\right)\left(z(u,v)-\lambda \right)z\left(u,v\right)\\ =0,(u,v)\in \eta ,\\ z\left(u,0\right)=z_{0}\left(u\right),u\in \Gamma_{i}=\left\{(u,0):u\in [0,1]\right\},\\ z\left(0,v\right)=\varphi_{0}\left(v\right),\forall \left(u,v\right)\in \Gamma_{l}=\left\{(u,v):u=0,v\in [0,K]\right\}\\ z\left(1,v\right)=\varphi_{1}\left(v\right),\forall \left(r,s\right)\in \Gamma_{u}=\left\{(u,v):u=1,v\in [0,K]\right\}, \end{array}\right.$$

where $\eta =\Omega_{r}\times \Omega_{v}=\left(0,1\right)\times \left(0,K\right]$, α≥1,θ≥0,λ∈(0,1), and $\partial \eta =\Gamma_{l}⋃\Gamma_{i}⋃\Gamma_{r}$, where $\Gamma_{l}$ is left boundary, $\Gamma_{i}$ is initial boundary and $\Gamma_{u}$ is right boundary. The functions $\varphi_{0}\left(v\right),\varphi_{1}\left(v\right)$ and $z_{0}\left(u\right)$ are presumed to be sufficiently smooth, bounded and independent of ε. If ε=1 and α=0 Eq. (1) becomes to Huxley equation [34]:

(2) $$\frac{\partial z}{\partial v}\left(u,v\right)-\frac{\partial^{2}z}{\partial u^{2}}\left(u,v\right)-\theta \left(1-z(u,v)\right)\left(z(u,v)-\lambda \right)z\left(u,v\right)=0.$$

Eq. (2) describes nerve pulse propagation in nerve fibers and wall motion in liquid crystals [13]. If θ=0 Eq. (1) becomes to Burgers’ equation at high Reynolds number [35]:

$$\frac{\partial z}{\partial v}\left(u,v\right)-\varepsilon \frac{\partial^{2}z}{\partial u^{2}}\left(u,v\right)+\alpha z\frac{\partial z}{\partial u}\left(u,v\right)=0.$$

This equation describes the propagation of shock waves in one-dimensional fluid flow and turbulence [13].

### Properties of the solutions of continuous problem

Lemma 1.1 **Maximum principle**

*If* $z\in C^{2,1}\left(\bar{\eta }\right)$ *,* ${z|}_{\partial \eta }\geq 0$ *and* ${£}_{\varepsilon }{z|}_{\eta }\geq 0$ *, then* ${z|}_{\bar{\eta }}\geq 0$ *.*

Proof Assume that there exists a point $\left(u^{*},v^{*}\right)\in \bar{\eta },$ such that $z\left(u^{*},v^{*}\right)=\min_{\bar{\eta }\left(u,v\right)}<0.$ From the suppositions, we have $(u^{*},v^{*})\in \partial \eta ,$ $\frac{\partial z}{\partial v}\left(u^{*},v^{*}\right)=\frac{\partial z}{\partial u}\left(u^{*},v^{*}\right)=0$ and $\frac{\partial^{2}z}{\partial u^{2}}\left(u^{*},v^{*}\right)\geq 0$. Thus,

$${£}_{\varepsilon }z\left(u^{*},v^{*}\right)=\frac{\partial z}{\partial v}\left(u^{*},v^{*}\right)-\varepsilon \frac{\partial^{2}z}{\partial u^{2}}\left(u^{*},v^{*}\right)+\alpha z\frac{\partial z}{\partial u}\left(u^{*},v^{*}\right)-\;\theta \left(1-z(u^{*},v^{*})\right)\left(z(u^{*},v^{*})-\lambda \right)z\left(u^{*},v^{*}\right)<0,$$

which contradicts to the supposition ${£}_{\varepsilon }z\geq 0$ on η and therefore, z(u,v)≥0 for all $\left(u,v\right)\in \bar{\eta }$. □

Lemma 1.2 **Continuous stability estimate**

*For all* ε>0 *, the solution* z(u,v) *of* Eq. (1) *is bounded by:*

$${∥z∥}_{\bar{\eta }}\leq K{∥z_{0}∥}_{\Gamma_{i}}+{∥z∥}_{\partial \eta }.$$

Proof Let ${ℑ}^{\pm }\left(u,v\right)$ be two comparison functions defined by:

$${ℑ}^{\pm }\left(u,v\right)=K{∥z_{0}∥}_{\Gamma_{i}}+{∥z∥}_{\partial \eta }\pm z\left(u,v\right),\forall \left(u,v\right)\in \bar{\eta }.$$

It is easily seen that ${ℑ}^{\pm }\left(u,v\right)\geq 0,\forall \left(u,v\right)\in \partial \eta$. Using the differential operator ${£}_{\varepsilon }$ in Eq. (1) on ${ℑ}^{\pm }\left(u,v\right)$, we have:

$${£}_{\varepsilon }{ℑ}^{\pm }\left(u,v\right)={∥z_{0}∥}_{\Gamma_{i}}\pm 0,\forall \left(u,v\right)\in \eta .$$

Thus, by using Lemma 1.1, we obtain, ${ℑ}^{\pm }\left(u,v\right)\geq 0,\forall \left(u,v\right)\in \bar{\eta }$, which gives the required result. □

### Description of the numerical scheme

#### Quasi-linearization technique

Let us rewrite Eq. (1) as

(3) $$\left\{ \begin{array}{c} \left(\frac{\partial z}{\partial v}-\varepsilon \frac{\partial^{2}z}{\partial u^{2}}\right)\left(u,v\right)=\zeta \left(u,v,z\left(u,v\right),z_{u}\left(u,v\right)\right),\left(u,v\right)\in \eta ,\\ z\left(u,0\right)=z_{0}\left(u\right),u\in \left[0,1\right],\\ z\left(0,v\right)=\varphi_{0}\left(v\right),z\left(1,t\right)=\varphi_{1}\left(v\right),v\in \left(0,K\right], \end{array}\right.$$

where $\zeta \left(u,v,z\left(u,v\right),z_{u}\left(u,v\right)\right)=-\alpha z\frac{\partial z}{\partial u}+\theta \left(1-z\right)\left(z-\lambda \right)z$ is the non-linear function of $u,v,z\left(u,v\right),z_{u}\left(u,v\right)$.

On applying the Newton-Raphson-Kantorovich technique on the nonlinear terms of Eq. (3), we obtain

(4) $$\begin{array}{ccc} \zeta \left(u,v,z^{\left(h+1\right)}\left(u,v\right),z_{u}^{\left(h+1\right)}\left(u,v\right)\right) & ≅ & \zeta \left(u,v,z^{\left(h\right)}\left(u,v\right),z_{u}^{\left(h\right)}\left(u,v\right)\right)+\;\left(z^{\left(h+1\right)}\left(u,v\right)-z^{\left(h\right)}\left(u,v\right)\right)\zeta_{u}{|}_{\left(u,vz^{\left(h\right)}\left(u,v\right),z_{u}^{\left(h\right)}\left(u,v\right)\right)}\\ & & +\;\left(z_{u}^{\left(h+1\right)}\left(u,v\right)-z_{u}^{\left(h\right)}\left(u,v\right)\right)\zeta_{z_{u}}{|}_{\left(u,v,z^{\left(h\right)}\left(u,v\right),z_{u}^{\left(h\right)}\left(u,v\right)\right)}+\cdots , \end{array}$$

where ${\left\{z^{\left(h\right)}\right\}}_{h=0}^{\infty }$ are a sequence of approximate solutions of $\zeta (u,v,z^{\left(h\right)}\left(u,v\right),z_{u}^{\left(h\right)}\left(u,v\right))$ and h is the iteration index.

Substituting Eq. (4) into Eq. (3), we have

(5) $$\left\{ \begin{array}{c} z_{v}^{\left(h+1\right)}\left(u,v\right)-\varepsilon z_{uu}^{\left(h+1\right)}\left(u,v\right)+\varpi \left(u,v\right)z_{u}^{\left(h+1\right)}\left(u,v\right)+q\left(u,v\right)z^{\left(h+1\right)}\left(u,v\right)=w\left(u,v\right),\\ z^{\left(h+1\right)}\left(u,0\right)=z_{0}\left(u\right),u\in \left[0,1\right],\\ z^{\left(h+1\right)}\left(0,v\right)=\varphi_{0}\left(v\right),v\in \left[0,K\right],\\ z^{\left(h+1\right)}\left(1,v\right)=\varphi_{1}\left(v\right),v\in \left[0,K\right], \end{array}\right.$$

where

$$\begin{array}{ccc} \varpi (u,v) & = & -\zeta_{z_{u}^{\left(h\right)}}{|}_{\left(u,v,z^{\left(h\right)}\left(u,v\right),z_{u}^{\left(h\right)}\left(u,v\right)\right)},\\ q(u,v) & = & -\zeta_{z^{\left(h\right)}}{|}_{\left(u,v,z^{\left(h\right)}\left(u,v\right),z_{u}^{\left(h\right)}\left(u,v\right)\right)},\\ w(u,v) & = & \zeta \left(u,v,z^{\left(h\right)}\left(u,v\right),z_{u}^{\left(h\right)}\left(u,v\right)\right)-z^{\left(h\right)}\zeta_{z^{\left(h\right)}}{|}_{\left(u,v,z^{\left(h\right)}\left(u,v\right),z_{u}^{\left(h\right)}\left(u,v\right)\right)}\;-z_{u}^{\left(h\right)}\zeta_{z_{u}^{\left(h\right)}}{|}_{\left(u,v,z^{\left(h\right)}\left(u,v\right),z_{u}^{\left(h\right)}\left(u,v\right)\right)}. \end{array}$$

### Temporal semi-discretization

We define the uniform mesh for the domain $\Omega_{v}$ as:

$$\Omega_{v}^{M}=\left\{v_{j}=j\tau =\;\tau =\frac{K}{M},\;j=0,1,2,\cdots ,M\right\}$$

and we approximated the temporal derivative term of Eq. (5) by employing implicit Euler method, which gives

(6) $$\left\{ \begin{array}{c} \left(1+\tau {£}_{\varepsilon }^{M}\right){\hat{z}}^{\left(h+1\right)}\left(u,v_{j+1}\right)={\hat{z}}^{\left(h+1\right)}\left(u,v_{j}\right),\\ {\hat{z}}^{\left(h+1\right)}\left(u,0\right)={\hat{z}}_{0}^{\left(h+1\right)}\left(u\right),u\in \left[0,1\right],\\ {\hat{z}}^{\left(h+1\right)}\left(0,v_{j+1}\right)=\varphi_{0}\left(v_{j+1}\right),j=0\left(1\right)M-1,\\ {\hat{z}}^{\left(h+1\right)}\left(1,v_{j+1}\right)=\varphi_{1}\left(v_{j+1}\right),j=0\left(1\right)M-1, \end{array}\right.$$

where

${£}_{\varepsilon }^{M}{\hat{z}}^{\left(h+1\right)}\left(u,v_{j+1}\right)=\left(-\varepsilon {\hat{z}}_{uu}^{\left(h+1\right)}+\varpi \left(u,v_{j+1}\right){\hat{z}}_{u}^{\left(h+1\right)}+q{\hat{z}}^{\left(h+1\right)}-w\right)\left(u,v_{j+1}\right)$.

Eq. (6) reveals boundary layer near u=1 and confesses a unique solution, since, $\varpi \left(u,v_{j+1}\right)\geq \varpi^{*}>0,\text{and}\;q\left(u,v_{j+1}\right)\geq q^{*}>0.u\in {\bar{\Omega }}_{u}$.

Lemma 1.3 *The local error estimate*$lte_{j+1}=z^{\left(h+1\right)}\left(u,v_{j+1}\right)-{\hat{z}}^{\left(h+1\right)}\left(u,v_{j+1}\right)$*in the temporal discretization at*(j+1)th*time level holds:*

$${∥lte_{j+1}∥}_{\infty }\leq C\tau^{2},$$

where C is a positive constant independent of ε and τ.

Proof Using Taylor’s series expansion on ${\hat{z}}^{\left(h+1\right)}\left(u,v_{j+1}\right)$, we have

$${\hat{z}}^{\left(h+1\right)}\left(u,v_{j+1}\right)={\hat{z}}^{\left(h+1\right)}\left(u,v_{j}\right)+\tau {\hat{z}}_{v}^{\left(h+1\right)}\left(u,v_{j}\right)+O\left({\left(\tau \right)}^{2}\right).$$

This implies

(7) $$\frac{{\hat{z}}^{\left(h+1\right)}\left(u,v_{j+1}\right)-{\hat{z}}^{\left(h+1\right)}\left(u,v_{j}\right)}{\tau }={\hat{z}}_{v}^{\left(h+1\right)}\left(u,v_{j}\right)+O\left(\tau \right).$$

Plugging Eq. (5) into Eq. (7) we have

$$\begin{array}{ccc} \frac{{\hat{z}}^{\left(h+1\right)}\left(u,v_{j+1}\right)-{\hat{z}}^{\left(h+1\right)}\left(u,v_{j}\right)}{\tau } & = & -\left(-\varepsilon {\hat{z}}_{uu}^{\left(h+1\right)}\left(u,v_{j}\right)+\varpi \left(u,v_{j}\right){\hat{z}}_{u}^{\left(h+1\right)}\left(u,v_{j}\right)\right)+\\ & & -\;\left(q\left(u,v_{j}\right){\hat{z}}^{\left(h+1\right)}\left(u,v_{j}\right)-w\left(u,v_{j}\right)\right)+O\left(\tau \right). \end{array}$$

This yields

(8) $$\left(1+\tau {£}_{\varepsilon }^{M}\right){\hat{z}}^{\left(h+1\right)}\left(u,v_{j+1}\right)-\tau w\left(u,v_{j}\right)+{\hat{z}}^{\left(h+1\right)}\left(u,v_{j}\right)=O{\left(\tau \right)}^{2}).$$

Subtracting Eq. (6) from Eq. (8), and the local truncation estimate at (j+1)th is the solution of a BVP

(9) $$\left(1+\tau {£}_{\varepsilon }^{M}\right)lte_{j+1}=O\left({\left(\tau \right)}^{2}\right),lte_{j+1}\left(0\right)=0,\;\text{and}\;\;lte_{j+1}\left(1\right)=0.$$

Thus, using the application maximum principle on Eq. (9), we obtain

$${∥lte_{j+1}∥}_{\infty }\leq C\tau^{2}.□$$

Lemma 1.4 *The global error estimate*$\left(gee_{j}\right)$*in the temporal direction of Eq.*(6)*holds:*

$${∥gee_{j}∥}_{\infty }\leq C\tau ,\;\forall \;j\leq K/\tau .$$

Proof Using the result in Lemma 1.3, we have

$$\begin{array}{ccc} {∥gge_{j}∥}_{\infty } & = & {∥\sum_{i=1}^{j}LTE_{i}∥}_{\infty },\;\;\;j\leq K/\tau \\ & \leq & {∥LTE_{1}∥}_{\infty }+{∥LTE_{2}∥}_{\infty }+{∥LTE_{3}∥}_{\infty }+\cdots +{∥e_{j}∥}_{\infty }\\ & \leq & Cj{\left(\tau \right)}^{2}\;\;\;\left(\text{by}\;\;\;\text{Lemma}\;3\right)\\ & \leq & C\left(j\tau \right)\left(\tau \right)\\ & \leq & CK\left(\tau \right)\;\left(j\tau \leq K\right)\\ & \leq & C\tau . \end{array}$$

 □

Lemma 1.5 *For all*ε>0*, the solution*${\hat{z}}^{\left(h+1\right)}\left(u\right)$*of the*Eq. (6)*and its derivatives hold the following bound:*

$${∥\frac{\partial^{n}{\hat{z}}^{\left(h+1\right)}\left(u,v_{j+1}\right)}{\partial u^{n}}∥}_{\left[0,1\right]}\leq C\left(1+\varepsilon^{-n}\exp\left(\frac{-(\varpi^{*}\left(1-u\right))}{\varepsilon }\right)\right),0\leq n\leq 4.$$

Proof See [6]. □

For the sake of simplicity Eq. (6) can be rewritten as:

(10) $$\left\{ \begin{array}{c} -\varepsilon Z_{uu}^{j+1}\left(u\right)+p\left(u\right)Z_{u}^{j+1}\left(u\right)+Q\left(u\right)Z^{j+1}\left(u\right)=g^{j+1}\left(u\right)\\ Z\left(u,0\right)=Z^{0}\left(u\right),u\in \left[0,1\right],\\ Z^{j+1}\left(0\right)=\varphi_{0}^{j+1},j=0(1)M-1,\\ Z^{j+1}\left(1\right)=\varphi_{1}^{j+1},j=0(1)M-1, \end{array}\right.$$

where $Z^{j}\left(u\right)={\hat{z}}^{\left(h+1\right)}\left(u,v_{j}\right)$, $Q\left(u\right)=\left(q(u,v_{\left(h+1\right)})+1/\tau \right)$, $p\left(u\right)=\varpi \left(u,v_{j+1}\right)$ and $g^{j+1}\left(u\right)={\hat{z}}^{\left(h+1\right)}\left(u,v_{j}\right)/\tau +\upsilon \left(u,v_{j+1}\right)$.

#### Spatial discretization

On ${\bar{\Omega }}_{u}^{N}={\left\{u_{i}\right\}}_{i=0}^{N}$, a piecewise-uniform Shishkin mesh is formulated as follows. Let ${\bar{\Omega }}_{u}^{N}$ is divided into [0,1−γ], and [1−γ,1] such that ${\bar{\Omega }}_{u}^{N}=\left[0,1-\gamma \right]\cup \left(1-\gamma ,1\right]$, parameter γ is separating the uniform meshes is given by

$$\gamma =\min\left(\frac{1}{2},\gamma_{0}\varepsilon \ln\left(N\right)\right),\;\;\;\;\;\gamma_{0}\geq \frac{1}{\varpi^{*}}.$$

The mesh [0,1] is given by:

$$u_{i}=\left\{ \begin{array}{cc} i\ell_{i}, & \text{for}\;\;i=0(1)N/2,\\ 1-\gamma +\left(i-\frac{N}{2}\right)\ell_{i}, & \text{for}\;\;\;i=N/2+1(1)N, \end{array}\right.$$

where

$$\ell_{i}=u_{i}-u_{i-1}=\left\{ \begin{array}{cc} \frac{2(1-\gamma )}{N}, & \text{for}\;\;\;i=1(1)N/2,\\ \frac{2\gamma }{N}, & \text{for}\;\;\;i=N/2+1(1)N. \end{array}\right.$$

Now, we fully discretized the problem (10) via a hybrid computational scheme which is based on the midpoint upwind method and cubic spline in tension method in the outside and the inside layer region, respectively.

#### Midpoint upwind method

Applying the midpoint upwind method on the outside layer region of Eq. (6) gives:

(11) $${£}_{mu}^{N,M}Z_{i}^{j+1}=\left\{ \begin{array}{c} -\varepsilon D_{u}^{+}D_{u}^{-}Z_{i}^{j+1}+p_{i-1/2}D_{u}^{-}Z_{i}^{j+1}+Q_{i-1/2}Z_{i-1/2}^{j+1}=g_{i-1/2}^{j+1},\\ Z^{j+1}\left(0\right)=\varphi_{0}^{j+1},\;j=1(1)M-1,\\ Z^{j+1}\left(1\right)=\varphi_{1}^{j+1},\;j=1(1)M-1, \end{array}\right.$$

where $D_{u}^{+}D_{u}^{-}\Theta_{i}^{j+1}=\frac{2}{\ell_{i}+\ell_{i-1}}\left(\frac{\Theta_{i+1}^{j+1}-\Theta_{i}^{j+1}}{\ell_{i}}-\frac{\Theta_{i}^{j+1}-\Theta_{i-1}^{j+1}}{\ell_{i-1}}\right),D_{u}^{-}\Theta_{i}^{j+1}=\frac{\Theta_{i}^{j+1}-\Theta_{i-1}^{j+1}}{\ell_{i}}$, ${£}_{mu}^{N,M}\Theta_{i}^{j+1}$ is the midpoint upwind difference operator, $p_{i-1/2}=\left(\frac{p_{i}+p_{i-1}}{2}\right)$, $Q_{i-1/2}=\left(\frac{Q_{i}+Q_{i-1}}{2}\right)$ and $g_{i-1/2}^{j+1}=\left(\frac{g_{i}^{j+1}+g_{i-1}^{j+1}}{2}\right)$.

Eq. (11) can be written in the form of systems of equations as:

(12) $${£}_{mu}^{N,M}Z_{i}^{j+1}=\vartheta_{i}^{-}Z_{i-1}^{j+1}+\vartheta_{i}^{0}Z_{i}^{j+1}+\vartheta_{i}^{+}Z_{i+1}^{j+1}=G_{i}^{j+1},i=1\left(1\right)N-1,j=0\left(1\right)M-1.$$

where

$$\begin{array}{ccc} \vartheta_{i}^{-} & = & \frac{-2\varepsilon }{\ell_{i-1}\left(\ell_{i}+\ell_{i-1}\right)}-\frac{p_{i-1/2}}{\ell_{i}}+\frac{Q_{i-1/2}}{2},\\ \vartheta_{i}^{0} & = & \frac{2\varepsilon }{\ell_{i}\ell_{i-1}}+\frac{p_{i-1/2}}{\ell_{i}}+\frac{Q_{i-1/2}}{2},\\ \vartheta_{i}^{+} & = & \frac{-2\varepsilon }{\ell_{i}\left(\ell_{i}+\ell_{i-1}\right)},\\ G_{i}^{j+1} & = & g_{i-1/2}^{j+1}. \end{array}$$

#### Cubic spline in tension method

We discritized the inside layer region of Eq. (10) via cubic spline in tension method as discussed below. A function $S^{j+1}\left(u,\rho \right)\in C^{2}\left[0,1\right]$ which interpolates $Z^{j+1}\left(u\right)$ at the mesh points $u_{i}$, i=0(1)N, depends on a parameter ρ>0 reduces to cubic spline in the interval [0,1] as ρ→0 is known as parametric cubic spline function. The spline function $S^{j+1}\left(u,\rho \right)=S^{j+1}\left(u\right)$ satisfying in $\left[u_{i},u_{i+1}\right]$, the differential equation

(13) $$\begin{array}{ccc} \frac{d^{2}S^{j+1}\left(u\right)}{d^{2}u}+\rho S^{j+1}\left(u\right) & = & \left[\frac{d^{2}S^{j+1}\left(u_{i}\right)}{du^{2}}+\rho S^{j+1}\left(u_{i}\right)\right]\left(\frac{u_{i+1}-u}{\ell }\right)\\ & & +\;\left[\frac{d^{2}S^{j+1}\left(u_{i+1}\right)}{du^{2}}+\rho S^{j+1}\left(u_{i+1}\right)\right]\left(\frac{u-u_{i}}{\ell }\right), \end{array}$$

where $S^{j+1}\left(u_{i}\right)=Z_{i}^{j+1}$ and ρ>0 is known to be cubic spline in tension. Using the result in [27], we obtain the following scheme:

(14) $$\sigma_{1}\ell_{i}M_{i+1}^{j+1}+\sigma_{2}\left(\ell_{i}+\ell_{i-1}\right)M_{i}^{j+1}+\sigma_{1}\ell_{i-1}M_{i-1}^{j+1}=\frac{Z_{i+1}^{j+1}-Z_{i}^{j+1}}{\ell_{i}}-\frac{Z_{i}^{j+1}-Z_{i-1}^{j+1}}{\ell_{i-1}},\;\text{for}\;i=1\left(1\right)N-1,$$

where $\sigma_{1}=\frac{1}{\sigma^{2}}\left(1-\frac{\sigma }{\sinh\sigma }\right),\sigma_{2}=\frac{1}{\sigma^{2}}\left(\sigma \coth\sigma -1\right)$, $\sigma =\ell \rho^{1/2}$ and $M_{k}=\frac{d^{2}S^{j+1}\left(u_{k}\right)}{du^{2}},k=i,i\pm 1$. For the choice of parameters $\sigma_{1}+\sigma_{2}=1/2$, Eq. (14) is stable and has potential for tackling the given differential problem.

Applying Taylor’s series expansion on Z about $u_{k}$, k=i±1, we have:

(15) $$Z^{j+1}\left(u_{i-1}\right)\approx Z^{j+1}\left(u_{i}\right)-\ell_{i-1}\frac{dZ^{j+1}\left(u_{i}\right)}{du}+\frac{\ell_{i-1}^{2}}{2}\frac{d^{2}Z^{j+1}\left(u_{i}\right)}{du^{2}},$$

(16) $$Z^{j+1}\left(u_{i+1}\right)\approx Z^{j+1}\left(u_{i}\right)+\ell_{i}\frac{dZ^{j+1}\left(u_{i}\right)}{du}+\frac{\ell_{i}^{2}}{2}\frac{d^{2}Z^{j+1}\left(u_{i}\right)}{du^{2}}.$$

Multiplying Eq. (15) by $\ell_{i}^{2}/\ell_{i-1}^{2}$ and then subtracting it from Eq. (16), we get an approximation for $\frac{dZ^{j+1}\left(u_{i}\right)}{du}$:

(17) $$\frac{dZ^{j+1}\left(u_{i}\right)}{du}\approx \frac{1}{\ell_{i}\ell_{i-1}\left(\ell_{i}+\ell_{i-1}\right)}\left(-\ell_{i}^{2}Z^{j+1}\left(u_{i-1}\right)+\left(\ell_{i}^{2}-\ell_{i-1}^{2}\right)Z^{j+1}\left(u_{i}\right)+\ell_{i-1}^{2}Z^{j+1}\left(u_{i+1}\right)\right).$$

Similarly, multiplying (15) by $\ell_{i}/\ell_{i-1}$ and then adding it to Eq. (16), we get an approximation for $\frac{d^{2}Z^{j+1}\left(u_{i}\right)}{du^{2}}$:

(18) $$\frac{d^{2}Z^{j+1}\left(u_{i}\right)}{du^{2}}\approx \frac{2}{\ell_{i}\ell_{i-1}\left(\ell_{i}+\ell_{i-1}\right)}\left(\ell_{i}Z^{j+1}\left(u_{i-1}\right)+\left(\ell_{i}+\ell_{i-1}\right)Z^{j+1}\left(u_{i}\right)+\ell_{i-1}Z^{j+1}\left(u_{i+1}\right)\right).$$

Inserting Eqs. (17) and (18) in $\frac{dZ^{j+1}\left(u_{i+1}\right)}{du}\approx \frac{dZ^{j+1}\left(u_{i}\right)}{dr}+\ell_{i}\frac{d^{2}Z^{j+1}\left(u_{i+1}\right)}{du^{2}}$ and $\frac{dZ^{j+1}\left(u_{i-1}\right)}{du}\approx \frac{dZ^{j+1}\left(u_{i}\right)}{du}+\ell_{i-1}\frac{d^{2}Z^{j+1}\left(\ell_{i+1}\right)}{du^{2}}$, we obtain

(19) $$\frac{dZ^{j+1}\left(u_{i-1}\right)}{du}\approx \frac{1}{\ell_{i}\ell_{i-1}\left(\ell_{i}+\ell_{i-1}\right)}\left(-\left(\ell_{i}^{2}+2\ell_{i}\ell_{i-1}\right)Z^{j+1}\left(u_{i-1}\right)+{\left(\ell_{i}+\ell_{i-1}\right)}^{2}Z^{j+1}\left(u_{i}\right)\right)\;-\;\frac{1}{\ell_{i}\ell_{i-1}\left(\ell_{i}+\ell_{i-1}\right)}\left(\ell_{i-1}^{2}Z^{j+1}\left(u_{i+1}\right)\right).$$

(20) $$\frac{dZ^{j+1}\left(u_{i+1}\right)}{du}\approx \frac{1}{\ell_{i}\ell_{i-1}\left(\ell_{i}+\ell_{i-1}\right)}\left(-\ell_{i}^{2}Z^{j+1}\left(u_{i-1}\right)-{\left(\ell_{i}+\ell_{i-1}\right)}^{2}Z^{j+1}\left(u_{i}\right)\right)+\frac{1}{\ell_{i}\ell_{i-1}\left(\ell_{i}+\ell_{i-1}\right)}\left(\left(\ell_{i}^{2}+2\ell_{i}\ell_{i-1}\right)Z^{j+1}\left(u_{i+1}\right)\right).$$

Eq. (6) at $u=u_{k}$ can be written as:

(21) $$-\varepsilon M_{k}+p_{k}\frac{dZ^{j+1}\left(u_{k}\right)}{du}+Q_{k}Z^{j+1}\left(u_{k}\right)=g^{j+1}\left(u_{k}\right),\;\;\;k=i,i\pm 1.$$

Substituting Eqs. ((17)–(20)) into Eq. (21) and substituting the obtained result scheme into Eq. (14) gives

(22) $${£}_{cs}^{N,M}=\phi_{i}^{-}Z_{i-1}^{j+1}+\phi_{i}^{0}Z_{i}^{j+1}+\phi_{i}^{+}Z_{i+1}^{j+1}=F_{i}^{j+1},i=1\left(1\right)N-1,j=0\left(1\right)M-1,$$

where ${£}_{cs}^{N,M}$ is the cubic spline in tension operator,

$$\begin{array}{ccc} \phi_{i}^{-} & = & -\frac{\varepsilon }{\ell_{i-1}\left(\ell_{i-1}+\ell_{i}\right)}-\sigma_{1}\frac{p_{i-1}\left(\ell_{i}+2\ell_{i-1}\right)}{{\left(\ell_{i}+\ell_{i-1}\right)}^{2}}-\sigma_{2}\frac{p_{i}\ell_{i}}{\ell_{i-1}\left(\ell_{i-1}+\ell_{i}\right)}\\ & & +\;\sigma_{1}\frac{p_{i+1}\ell_{i}^{2}}{\ell_{i-1}{\left(\ell_{i}+\ell_{i-1}\right)}^{2}}+\sigma_{1}Q_{i-1}\frac{\ell_{i-1}}{\left(\ell_{i-1}+\ell_{i}\right)},\\ \phi_{i}^{0} & = & \frac{\varepsilon (\ell_{i}+\ell_{i-1})}{\ell_{i}\ell_{\ell_{i-1}}\left(\ell_{i-1}+\ell_{i}\right)}+\sigma_{1}\frac{p_{i-1}}{\ell_{i}}+\sigma_{2}\frac{p_{i}\left(\ell_{i}-\ell_{i-1}\right)}{\ell_{i}\ell_{i-1}}-\sigma_{1}\frac{p_{i+1}}{h_{i-1}}+\sigma_{2}Q_{i},\\ \phi_{i}^{+} & = & -\frac{\varepsilon }{\ell_{i}\left(\ell_{i-1}+\ell_{i}\right)}-\sigma_{1}\frac{p_{i-1}\ell_{i-1}^{2}}{\ell_{i}{\left(\ell_{i-1}+\ell_{i}\right)}^{2}}+\sigma_{2}\frac{p_{i}\ell_{i-1}}{\ell_{i}\left(\ell_{i-1}+\ell_{i}\right)}\\ & & +\;\sigma_{1}\frac{p_{i+1}\left(\ell_{i-1}+2\ell_{i}\right)}{{\left(\ell_{i}+\ell_{i-1}\right)}^{2}}+\sigma_{1}Q_{i+1}\frac{\ell_{i}}{\left(\ell_{i-1}+\ell_{i}\right)}.\\ F_{i}^{j+1} & = & \sigma_{1}\frac{\ell_{i-1}}{\left(\ell_{i-1}+\ell_{i}\right)}g_{i-1}^{j+1}+\sigma_{2}g_{i}^{j+1}+\sigma_{1}\frac{\ell_{i}}{\left(\ell_{i-1}+\ell_{i}\right)}g_{i+1}^{j+1}. \end{array}$$

The totally discrete scheme takes the form:

(23) $${£}_{hyb}^{N,M}Z_{i}^{j+1}=\left\{ \begin{array}{ccc} {£}_{mu}^{N,M}Z_{i}^{j+1}=g_{i-1/2}^{j+1}, & \text{for} & i=1(1)N/2,\\ {£}_{cs}^{N,M}Z_{i}^{j+1}=g_{i}^{j+1}, & \text{for} & i=N/2+1(1)N,\\ Z_{0}^{j+1}=\varphi_{0}^{j+1}, & \text{for} & j=0(1)M-1,\\ Z_{N}^{j+1}=\varphi_{1}^{j+1}, & \text{for} & j=0(1)M-1,\\ Z_{i}^{0}=Z^{0}\left(u_{i}\right), & \text{for} & i=0(1)N. \end{array}\right.$$

After the rearrangement of Eq. (23), we have

(24) $$\psi_{i}^{-}Z_{i-1}^{j+1}+\psi_{i}^{0}Z_{i}^{j+1}+\psi_{i}^{+}Z_{i+1}^{j+1}=H_{i}^{j+1},i=0\left(1\right)N,j=0\left(1\right)M,$$

where

$$\psi_{i}^{-}=\left\{ \begin{array}{c} \frac{-2\varepsilon }{\ell_{i}\left(\ell_{i}+\ell_{i+1}\right)}-\frac{p_{i-1/2}}{\ell_{i+1}}+\frac{Q_{i-1/2}}{2},\;\;\;\;\;\;\;\;\;\;\;\;\;\;\;\;\;\;\;\;\;\;\;\;\;\;\;\text{for}\;\;i=0\left(1\right)N/2,\\ -\frac{\varepsilon }{\ell_{i-1}\left(\ell_{i-1}+\ell_{i}\right)}-\sigma_{1}\frac{p_{i-1}\left(\ell_{i}+2\ell_{i-1}\right)}{{\left(\ell_{i}+\ell_{i-1}\right)}^{2}}-\sigma_{2}\frac{p_{i}\ell_{i}}{\ell_{i-1}\left(\ell_{i-1}+\ell_{i}\right)}+\sigma_{1}\frac{p_{i+1}\ell_{i}^{2}}{\ell_{i-1}{\left(\ell_{i}+\ell_{i-1}\right)}^{2}}\\ +\sigma_{1}Q_{i-1}\frac{\ell_{i-1}}{\left(\ell_{i-1}+\ell_{i}\right)},\;\;\;\;\;\;\;\;\;\;\;\;\;\;\;\;\;\;\;\;\;\;\;\;\;\;\;\;\;\;\;\;\;\;\;\;\;\;\;\;\;\text{for}\;j=N/2+1\left(1\right)N. \end{array}\right.$$

$$\psi_{i}^{0}=\left\{ \begin{array}{c} \frac{2\varepsilon }{\ell_{i}\ell_{i+1}}+\frac{p_{i-1/2}}{\ell_{i}}+\frac{Q_{i-1/2}}{2},\;\;\;\;\;\;\;\;\;\;\;\;\;\;\;\;\;\;\;\;\;\;\;\;\;\;\;\;\;\;\;\;\;\;\;\;\text{for}\;i=0\left(1\right)N/2,\\ \frac{\varepsilon (\ell_{i}+\ell_{i-1})}{\ell_{i}\ell_{\ell_{i-1}}\left(\ell_{i-1}+\ell_{i}\right)}+\sigma_{1}\frac{p_{i-1}}{\ell_{i}}+\sigma_{2}\frac{p_{i}\left(\ell_{i}-\ell_{i-1}\right)}{\ell_{i}\ell_{i-1}}-\sigma_{1}\frac{p_{i+1}}{\ell_{i-1}}+\sigma_{2}Q_{i},\text{for}\;i=N/2+1\left(1\right)N. \end{array}\right.$$

$$\psi_{i}^{+}=\left\{ \begin{array}{c} \frac{-2\varepsilon }{\ell_{i+1}\left(\ell_{i}+\ell_{i+1}\right)},\;\;\;\;\;\;\;\;\;\;\;\;\;\;\;\;\;\;\;\;\;\;\;\;\;\;\;\;\;\;\;\;\;\;\;\;\;\;\;\;\;\;\;\;\;\;\;\;\;\;\text{for}\;\;\;i=0\left(1\right)N/2,\\ -\frac{\varepsilon }{\ell_{i}\left(\ell_{i-1}+\ell_{i}\right)}-\sigma_{1}\frac{p_{i-1}\ell_{i-1}^{2}}{\ell_{i}{\left(\ell_{i-1}+\ell_{i}\right)}^{2}}+\sigma_{2}\frac{p_{i}\ell_{i-1}}{\ell_{i}\left(\ell_{i-1}+\ell_{i}\right)}+\sigma_{1}\frac{p_{i+1}\left(\ell_{i-1}+2\ell_{i}\right)}{{\left(\ell_{i}+\ell_{i-1}\right)}^{2}}\\ +\sigma_{1}Q_{i+1}\frac{\ell_{i}}{\left(\ell_{i-1}+\ell_{i}\right)},\;\;\;\;\;\;\;\;\;\;\;\;\;\;\;\;\;\;\;\;\;\;\;\;\;\;\;\;\;\;\;\;\;\;\;\;\;\;\;\;\;\;\;\text{for}\;i=N/2+1\left(1\right)N. \end{array}\right.$$

$$H_{i}^{j+1}=\left\{ \begin{array}{c} g_{i-1/2}^{j+1},\;\;\;\;\;\;\text{for}\;\;i=0\left(1\right)N/2,\\ F_{i}^{j+1},\;\;\;\;\;\;\;\text{for}\;\;i=N/2+1\left(1\right)N. \end{array}\right.$$

### Error analysis

ε−uniform convergence analysis of the proposed numerical scheme is the primary focus of this section. We analyzed the ε−uniform convergence of the proposed method divided into two cases as follows.

Case (i). For i=N/2+1(1)N, we have the truncation error as:

(25) $$\begin{array}{ccc} T_{i} & = & \psi_{i}^{-}Z^{j+1}\left(u_{i-1}\right)+\psi_{i}^{0}Z^{j+1}\left(u_{i}\right)+\psi_{i}^{+}Z^{j+1}\left(u_{i+1}\right)-\sigma_{1}\frac{\ell_{i-1}}{\left(\ell_{i}+\ell_{i-1}\right)}g^{j+1}\left(u_{i-1}\right)\\ & & -\;\sigma_{2}g^{j+1}\left(u_{i}\right)-\sigma_{1}\frac{\ell_{i}}{\left(\ell_{i}+\ell_{i-1}\right)}g^{j+1}\left(u_{i+1}\right),\;\;\;i=1\left(1\right)N-1,j=0\left(1\right)M-1. \end{array}$$

Using the Eq. (6) for $g^{j+1}\left(u_{i+1}\right),g^{j+1}\left(u_{i}\right)$ and $g^{j+1}\left(u_{i+1}\right)$ in Eq. (25), we get

(26) $$\begin{array}{ccc} T_{i} & = & \psi_{i}^{-}Z^{j+1}\left(u_{i-1}\right)+\psi_{i}^{0}Z^{j+1}\left(u_{i}\right)+\psi_{i}^{+}Z^{j+1}\left(u_{i+1}\right)\\ & & -\;\sigma_{1}\frac{\ell_{i-1}}{\left(\ell_{i}+\ell_{i-1}\right)}\left\{-\varepsilon \frac{d^{2}Z^{j+1}\left(u_{i-1}\right)}{du^{2}}+p\left(u_{i-1}\right)\frac{dZ^{j+1}\left(u_{i-1}\right)}{du}+Q\left(u_{i-1}\right)Z^{j+1}\left(u_{i-1}\right)\right\}\\ & & -\;\sigma_{2}\left\{-\varepsilon \frac{d^{2}Z^{j+1}\left(u_{i}\right)}{du^{2}}+p\left(u_{i}\right)\frac{dZ^{j+1}\left(u_{i}\right)}{du}+Q\left(u_{i}\right)Z^{j+1}\left(u_{i}\right)\right\}\\ & & -\;\sigma_{1}\frac{\ell_{i}}{\left(\ell_{i}+\ell_{i-1}\right)}\left\{-\varepsilon \frac{d^{2}Z^{j+1}\left(u_{i+1}\right)}{du^{2}}+p\left(u_{i+1}\right)\frac{dZ^{j+1}\left(u_{i+1}\right)}{du}+Q\left(u_{i+1}\right)Z^{j+1}\left(u_{i+1}\right)\right\},\\ i & = & 1(1)N-1,j=0(1)M-1. \end{array}$$

On applying Taylor’s series expansion on $Z^{j+1}\left(u_{i-1}\right)$ and $Z^{j+1}\left(u_{i+1}\right)$ in spatial variable, we have

(27) $$Z^{j+1}\left(u_{i-1}\right)\approx Z^{j+1}\left(u_{i}\right)-\ell_{i-1}\frac{dZ^{j+1}\left(u_{i}\right)}{du}+\frac{\ell_{i-1}^{2}}{2}\frac{d^{2}Z^{j+1}\left(u_{i}\right)}{du^{2}}-\frac{\ell_{i-1}^{3}}{6}\frac{d^{3}Z^{j+1}\left(u_{i}\right)}{du^{3}}+\;\frac{\ell_{i-1}^{4}}{24}\frac{d^{4}Z^{j+1}\left(u_{i}\right)}{du^{4}}+\cdots ,$$

(28) $$Z^{j+1}\left(u_{i+1}\right)\approx Z^{j+1}\left(u_{i}\right)+\_i\frac{dZ^{j+1}\left(u_{i}\right)}{du}+\frac{\ell_{i}^{2}}{2}\frac{d^{2}Z^{j+1}\left(u_{i}\right)}{du^{2}}+\frac{\ell_{i}^{3}}{6}\frac{d^{3}Z^{j+1}\left(\ell_{i}\right)}{du^{3}}+\frac{\ell_{i}^{4}}{24}\frac{d^{4}Z^{j+1}\left(u_{i}\right)}{du^{4}}+\cdots ,$$

(29) $$\frac{dZ^{j+1}\left(u_{i-1}\right)}{du}\approx \frac{dZ^{j+1}\left(u_{i}\right)}{du}-\ell_{i-1}\frac{d^{2}Z^{j+1}\left(u_{i}\right)}{du^{2}}+\frac{\ell_{i-1}^{2}}{2}\frac{d^{3}Z^{j+1}\left(u_{i}\right)}{du^{3}}-\frac{\ell_{i-1}^{3}}{6}\frac{d^{4}Z^{j+1}\left(u_{i}\right)}{du^{4}}+\cdots ,$$

(30) $$\frac{dZ^{j+1}\left(u_{i+1}\right)}{du}\approx \frac{dZ^{j+1}\left(u_{i}\right)}{du}+\ell_{i}\frac{d^{2}Z^{j+1}\left(u_{i}\right)}{du^{2}}+\frac{\ell_{i}^{2}}{2}\frac{d^{3}Z^{j+1}\left(u_{i}\right)}{du^{3}}+\frac{\ell_{i}^{3}}{6}\frac{d^{4}Z^{j+1}\left(u_{i}\right)}{du^{4}}+\cdots ,$$

(31) $$\frac{d^{2}Z^{j+1}\left(u_{i-1}\right)}{du^{2}}\approx \frac{d^{2}Z^{j+1}\left(u_{i}\right)}{du^{2}}-\ell_{i-1}\frac{d^{3}Z^{j+1}\left(u_{i}\right)}{du^{3}}+\frac{\ell_{i-1}^{2}}{2}\frac{d^{4}Z^{j+1}\left(u_{i}\right)}{du^{4}}+\cdots ,$$

(32) $$\frac{d^{2}Z^{j+1}\left(u_{i+1}\right)}{du^{2}}\approx \frac{d^{2}Z^{j+1}\left(u_{i}\right)}{du^{2}}+\ell_{i}\frac{d^{3}Z^{j+1}\left(u_{i}\right)}{du^{3}}+\frac{\ell_{i}^{2}}{2}\frac{d^{4}Z^{j+1}\left(u_{i}\right)}{du^{4}}+\cdots .$$

Substituting (Eqs. (27)–(32)) into Eq. (26), we get

(33) $$T_{i}=\chi_{1,i}Z^{j+1}\left(u_{i}\right)+\chi_{2,i}\frac{dZ^{j+1}\left(u_{i}\right)}{du}+\chi_{3,i}\frac{d^{2}Z^{j+1}\left(u_{i}\right)}{du^{2}}+\chi_{4,i}\frac{d^{3}Z^{j+1}\left(u_{i}\right)}{du^{3}}+\chi_{5,i}\frac{d^{4}Z^{j+1}\left(u_{i}\right)}{du^{4}}+\cdots ,$$

where

$$\begin{array}{ccc} \chi_{1,i} & = & \psi_{i}^{-}+\psi_{i}^{0}+\psi_{i}^{+}-\sigma_{1}\frac{\ell_{i-1}}{\left(\ell_{i}+\ell_{i-1}\right)}Q_{i-1}-\sigma_{2}Q_{i}-\sigma_{1}\frac{\ell_{i}}{\left(\ell_{i}+\ell_{i-1}\right)}Q_{i+1},\\ \chi_{2,i} & = & -\ell_{i-1}\psi^{-}+\ell_{i}\psi^{+}-\sigma_{1}\frac{\ell_{i-1}^{2}}{\left(\ell_{i}+\ell_{i-1}\right)}Q_{i-1}-\sigma_{2}p_{i}-\sigma_{1}\frac{\ell_{i}}{\left(\ell_{i}+\ell_{i-1}\right)}p_{i+1}-\sigma_{1}\frac{\ell_{i}^{2}}{\left(\ell_{i}+\ell_{i-1}\right)}Q_{i+1},\\ \chi_{3,i} & = & -\ell_{i-1}^{2}\psi_{i}^{-}+\frac{\ell_{i}^{2}}{2}\psi_{i}^{+}+\varepsilon \sigma_{1}\frac{\ell_{i-1}}{\left(\ell_{i}+\ell_{i-1}\right)}+\sigma_{1}\frac{\ell_{i+1}^{2}}{\left(\ell_{i}+\ell_{i-1}\right)}p_{i-1}-\sigma_{1}\frac{\ell_{i-1}^{3}}{2\left(\ell_{i}+\ell_{i-1}\right)}Q_{i-1}-\sigma_{1}\varepsilon \\ & & -\;\sigma_{1}\frac{\ell_{i}^{2}}{\left(\ell_{i}+\ell_{i-1}\right)}p_{i+1}+\varepsilon \sigma_{1}\frac{\ell_{i}}{\left(\ell_{i}+\ell_{i-1}\right)}-\sigma_{1}\frac{\ell_{i}^{3}}{2\left(\ell_{i}+\ell_{i-1}\right)}Q_{i+1},\\ \chi_{4,i} & = & -\frac{\ell_{i-1}^{3}}{6}\psi_{i}^{-}+\frac{\ell_{i}^{3}}{6}\psi_{i}^{+}-\varepsilon \sigma_{1}\frac{\ell_{i-1}^{2}}{\left(\ell_{i}+\ell_{i-1}\right)}-\sigma_{1}\frac{\ell_{i-1}^{3}}{2\left(\ell_{i}+\ell_{i-1}\right)}p_{i-1}+\sigma_{1}\frac{\ell_{i-1}^{4}}{6\left(\ell_{i}+\ell_{i-1}\right)}Q_{i-1}\\ & & +\;\varepsilon \sigma_{1}\frac{\ell_{i}^{2}}{\left(\ell_{i}+\ell_{i-1}\right)}-\sigma_{1}\frac{\ell_{i}^{2}}{2\left(\ell_{i}+\ell_{i-1}\right)}p_{i+1}-\sigma_{1}\frac{\ell_{i}^{4}}{6\left(\ell_{i}+\ell_{i-1}\right)}Q_{i+1},\\ \chi_{5,i} & = & \frac{\ell_{i-1}^{4}}{24}\psi_{i}^{-}++\frac{\ell_{i}^{4}}{24}\psi_{i}^{+}+\varepsilon \sigma_{1}\frac{\ell_{i-1}^{3}}{2\left(\ell_{i}+\ell_{i-1}\right)}+\sigma_{1}\frac{\ell_{i-1}^{4}}{6\left(\ell_{i}+\ell_{i-1}\right)}p_{i-1}-\sigma_{1}\frac{\ell_{i-1}^{5}}{24\left(\ell_{i}+h_{i-1}\right)}Q_{i-1}\\ & & +\;\varepsilon \sigma_{1}\frac{\ell_{i}^{3}}{2\left(\ell_{i}+\ell_{i-1}\right)}-\sigma_{1}\frac{\ell_{i}^{4}}{6\left(\ell_{i}+\ell_{i-1}\right)}p_{i+1}-\sigma_{1}\frac{\ell_{i}^{5}}{24\left(\ell_{i}+\ell_{i-1}\right)}Q_{i+1}. \end{array}$$

From the simplification of Eq. (33), we obtain

(34) $$\chi_{1,i}=\chi_{2,i}=0.$$

Using $\sigma_{1}+\sigma_{2}=\frac{1}{2}$ in the above expressions, we obtain

(35) $$\left\{ \begin{array}{c} \chi_{3,i}=\chi_{4,i}=0,\\ \chi_{5,i}=-\varepsilon \left(\frac{1}{24}-\frac{\sigma_{1}}{2}\right)\left(\frac{\ell_{i-1}^{3}+\ell_{i}^{3}}{\ell_{i-1}+\ell_{i}}\right). \end{array}\right.$$

Substitution of (Eqs. (34)–(35)) into Eq. (33) yields

(36) $$T_{i}=-\varepsilon \left(\frac{1}{24}-\frac{\sigma_{1}}{2}\right)\left(\frac{\ell_{i-1}^{3}+\ell_{i}^{3}}{\ell_{i-1}+\ell_{i}}\right)\frac{d^{4}Z^{j+1}\left(u_{i}\right)}{du^{4}}+O\left(\left(\tau \right)+N^{-3}\right).$$

Case(ii) For i=1(1)N/2, proceeding in a similar way of case(i), we obtained the truncation error as

(37) $$T_{i}=-\varepsilon \left(\frac{\ell_{i-1}^{3}+\ell_{i}^{3}}{\ell_{i-1}+\ell_{i}}\right)\frac{d^{4}Z^{j+1}\left(u_{i}\right)}{du^{4}}+O\left(\left(\tau \right)+N^{-3}\right).$$

Theorem 1.6 *Let*$z(u_{i},v_{j+1})$*be the solution of*(5)*and*$Z_{i}^{j+1}$*be the approximation to the solution of*(24)*. Then the error bound satisfies:*

$$\max_{0\leq i,j\leq N,M}\left|z\left(u_{i},v_{j+1}\right)-Z_{i}^{j+1}\right|\leq C\left(\tau +N^{-2}\ln^{3}N\right).$$

Proof To estimate the error bound, we consider the following two cases:

Case 1: When γ=1/2, $\ell_{i}=1/N$ and $W\left(\varepsilon /p^{*}\right)\ln N\geq 1/2$, which gives $\varepsilon^{-1}\leq C\ln N$. By using Lemma 1.5 in Eqs. ((36)–(37)), we obtain

(38) $$\left|T_{i}\right|\leq C\left(\tau +N^{-2}\ln^{3}N\right).$$

Case 2: When $\gamma =W\left(\varepsilon /p^{*}\right)\ln N$, we have

$$\ell_{i}=\left\{ \begin{array}{c} 2\left(1-\gamma \right)\;\;\text{in}\;\left[0,1-\gamma \right],\\ 2\gamma /N\;\;\;\text{in}\;\;[1-\gamma ,1]. \end{array}\right.$$

For the case 1≤i≤N/2, the region is outside layer (or regular) region i.e[0,1−γ]. From Lemma 1.5 we get

(39) $$\left|\frac{d^{\left(k\right)}Z_{i}^{j+1}}{du^{\left(k\right)}}\right|\leq C.$$

Using Eq. (39) into (37) we obtain

(40) $$\left|T_{i}\right|\leq C\left(\tau +N^{-2}\right).$$

For the case N/2<i≤N, the region is inside layer (or singular) region i.e[1−γ,1], we obtain $\ell =2\gamma /N=2W\left(\varepsilon /p^{*}\right)\ln N$, which gives $\ell /\varepsilon =CN^{-1}\ln N$.

From Eq. (36), we obtain

(41) $$\left|T_{i}\right|\leq C\left(\tau +N^{-2}\ln^{2}N\right).$$

Combing (Eqs. (38)–(41)), we obtain the required bound

(42) $$\max_{0\leq i,j\leq N,M}\left|z\left(u_{i},v_{j+1}\right)-Z_{i}^{j+1}\right|\leq C\left(\tau +N^{-2}\ln^{3}N\right).$$

 □

### Numerical examples and results

Three examples are presented in this section to ensure that the proposed scheme is applicable and efficient. In all cases, we did numerical experimentations by using $\sigma_{1}=1e-02$ and $\sigma_{2}=4.9e-01$. Since the exact solutions of these examples do not exist, we use the double mesh principle to compute the maximum point-wise absolute errors [3]:

$$E_{\varepsilon }^{N,M}=\max_{i=1(1)N-1,j=1(1)M-1}\left|Z_{i,j}^{N,M}-Z_{i,j}^{2N,2M}\right|,$$

where $Z_{i,j}^{N,M}$ and $Z_{i,j}^{2N,2M}$ are calculated numerical solutions obtained on the mesh $\eta^{N,M}=\Omega_{u}^{N}\times \Omega_{v}^{M}$ and $\eta^{2N,2M}=\Omega_{u}^{2N}\times \Omega_{v}^{2M}$ respectively. The ε−uniform maximum absolute errors ($E^{N,M}$), order of convergence $\left(\wedge_{\varepsilon }^{N,M}\right)$ and ε−uniform order of convergence $\left(\wedge^{N,M}\right)$ are calculated using

$$E^{N,M}=\max\left\{E_{\varepsilon }^{N,M}\right\},\wedge_{\varepsilon }^{N,M}=\log_{2}\left(\frac{E_{\varepsilon }^{N,M}}{E_{\varepsilon }^{2N,2M}}\right)\text{and}\;\wedge^{N,M}=\log_{2}\left(\frac{E^{N,M}}{E^{2N,2M}}\right),$$

respectively.

Example 1.7 Consider the following SPBHE:

$$\left\{ \begin{array}{c} \frac{\partial z(u,v)}{\partial v}-\varepsilon \frac{\partial^{2}z\left(u,v\right)}{\partial u^{2}}+z\left(u,v\right)\frac{\partial z(u,v)}{\partial u}-\left(1-z(u,v)\right)\left(z(u,v)-0.5\right)z\left(u,v\right)=0,\left(u,v\right)\in \eta ,\\ z\left(u,0\right)=u\left(1-u^{2}\right),u\in \Omega_{u},\\ z\left(0,v\right)=0=z\left(1,v\right),v\in \Omega_{v}. \end{array}\right.$$

Example 1.8 Consider the following SPBHE:

$$\left\{ \begin{array}{c} \frac{\partial z(u,v)}{\partial v}-\varepsilon \frac{\partial^{2}z\left(u,v\right)}{\partial u^{2}}+z\left(u,v\right)\frac{\partial z(u,v)}{\partial u}=0,\left(u,v\right)\in \eta ,\\ z\left(u,0\right)=u\left(1-u^{2}\right),u\in \Omega_{u},\\ z\left(0,v\right)=0=z\left(1,v\right),v\in \Omega_{v}. \end{array}\right.$$

Example 1.9 Consider the following SPBHE:

$$\left\{ \begin{array}{c} \frac{\partial z(u,v)}{\partial v}-\varepsilon \frac{\partial^{2}z\left(u,v\right)}{\partial u^{2}}+z\left(u,v\right)\frac{\partial z(u,v)}{\partial u}=\left(1-z(u,v)\right)\left(z(u,v)-0.5\right)z\left(u,v\right),\left(u,v\right)\in \eta ,\\ z\left(u,0\right)=\sin\left(\pi u\right),u\in \Omega_{u},\\ z\left(0,u\right)=0=z\left(1,u\right),v\in \Omega_{u}. \end{array}\right.$$

**Fig. 1:**
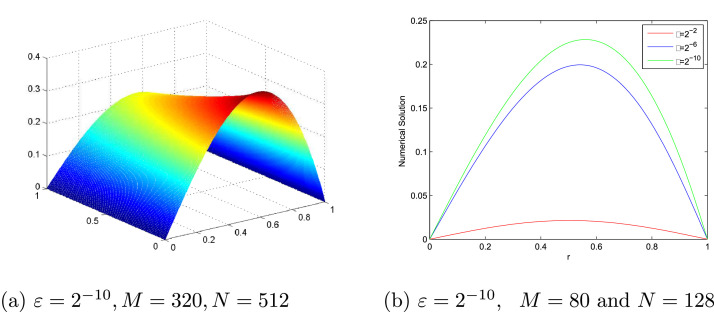
(a). Solution profile of Example 1.7, (b). Effect of ε on solution of Example 1.7.

**Fig. 2:**
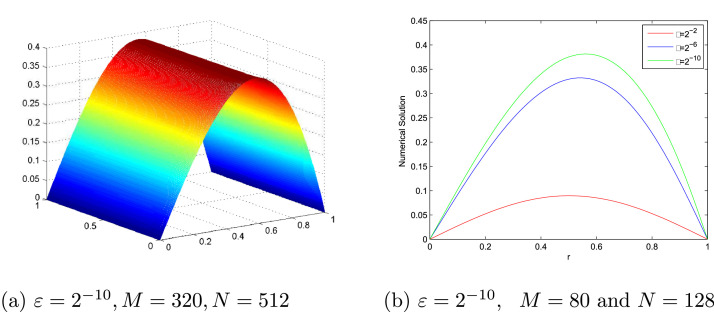
(a). Solution profile of Example 1.8, (b). Effect of ε on solution of Example 1.8.

**Fig. 3:**
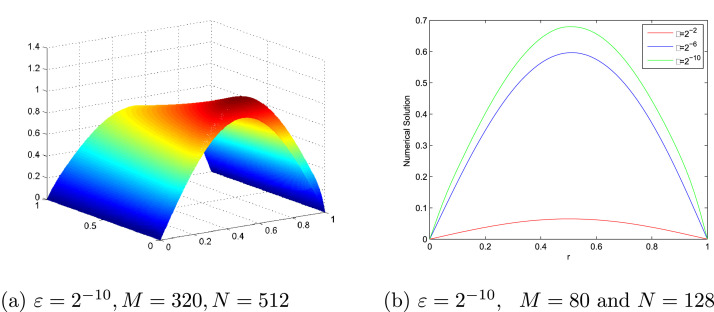
(a). Solution profile of Example 1.9, (b). Effect of ε on solution of Example 1.9.

**Fig. 4:**
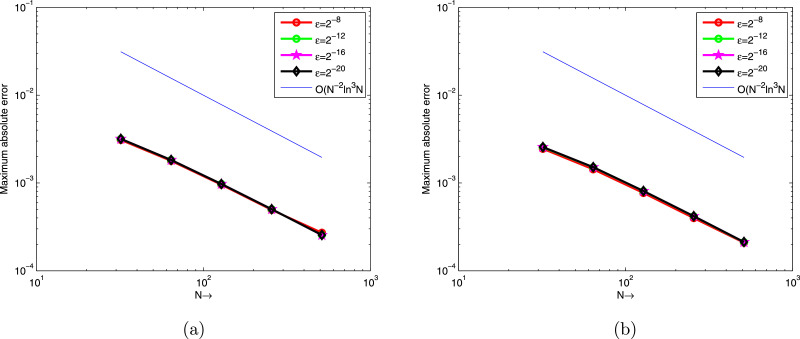
The maximum absolute error via loglog plot at various values of ε for Examples 1.7 and 1.8.

Tables 1 and 2 present a comparison of the $E_{\varepsilon }^{N,M},E^{N,M},\wedge_{\varepsilon }^{N,M}$ and $\wedge^{N,M}$ between the proposed scheme and the scheme in [17] at various values of N,M and ε for Examples 1.7 and 8, respectively. These show the accuracy of the proposed scheme. $E_{\varepsilon }^{N,M},E^{N,M},\wedge_{\varepsilon }^{N,M}$ and $\wedge^{N,M}$ comparison between the proposed scheme and the scheme in [13] at various values of N,M and ε for Example 1.9 is tabulated in Table 3. It can be observed that the proposed scheme has more accurate results than results in [13]. Fig. 1, Fig. 2, Fig. 3 display the numerical solutions for Examples 1.7–9. These figures show as ε→0 the width of the boundary layer increases and a strong boundary layer is created near u=1. Fig. 4 shows the log-log plot of the maximum absolute errors. It can be seen that the error decreases as N increases.

**Table 1:** Comparison of $E_{\varepsilon }^{N,M},E^{N,M}$, $\wedge_{\varepsilon }^{N,M}$, and $\wedge^{N,M}$ for Example 1.7 of the proposed scheme with results in [17].

ε↓N→	16	32	64	128	256	512
M→	10	20	40	80	160	320
Present method						
$2^{-8}$	4.2983e-03	3.0953e-03	1.7892e-03	9.5647e-04	4.9391e-04	2.7101e-04
	4.7369e-01	7.9076e-01	9.0352e-01	9.5347e-01	8.6590e-01	
$2^{-10}$	4.5326e-03	3.1670e-03	1.8173e-03	9.6932e-04	5.0036e-04	2.5406e-04
	5.1722e-01	8.0132e-01	9.0675e-01	9.5401e-01	9.7780e-01	
$2^{-12}$	4.5464e-03	3.1733e-03	1.8251e-03	9.7341e-04	5.0210e-04	2.5493e-04
	5.1874e-01	7.9801e-01	9.0686e-01	9.7787e-01	9.7787e-01	
$2^{-14}$	4.5469e-03	3.1783e-03	1.8273e-03	9.7423e-04	5.0251e-04	2.5513e-04
	5.1663e-01	7.9854e-01	9.0738e-01	9.5511e-01	9.7792e-01	
$2^{-16}$	4.5482e-03	3.1811e-03	1.8278e-03	9.7435e-04	5.0260e-04	2.5519e-04
	5.1577e-01	7.9942e-01	9.0760e-01	9.5503e-01	9.7784e-01	
$2^{-18}$	4.5487e-03	3.1823e-03	1.8276e-03	9.7448e-04	5.0262e-04	2.5520e-04
	5.1538e-01	8.0012e-01	9.0725e-01	9.5516e-01	9.7784e-01	
$2^{-20}$	4.5488e-03	3.1826e-03	1.8275e-03	9.7445e-04	5.0263e-04	2.5521e-04
	5.1528e-01	8.0033e-01	9.0721e-01	9.5509e-01	9.7781e-01	
$2^{-22}$	4.5488e-03	3.1827e-03	1.8275e-03	9.7443e-04	5.0264e-04	2.5521e-04
	5.1528e-01	8.0038e-01	9.0724e-01	9.5503e-01	9.7784e-01	
$2^{-24}$	4.5488e-03	3.1827e-03	1.8275e-03	9.7443e-04	5.0264e-04	2.5521e-04
	5.1528e-01	8.0038e-01	9.0724e-01	9.5503e-01	9.7784e-01	
$E^{N,M}$	4.5488e-03	3.1827e-03	1.8275e-03	9.7443e-04	5.0264e-04	2.5521e-04
$\wedge^{N,M}$	5.1528e-01	8.0038e-01	9.0724e-01	9.5503e-01	9.7784e-01	
Results in [17] using the adaptive grid method.						
$E^{N,M}$	–	2.5614e-01	2.1031e-01	1.3406e-01	8.5618e-02	4.8834e-02
$\wedge^{N,M}$	–	2.8442e-01	6.4964e-01	6.4689e-01	8.1003e-01	
Results in [17] using the Shishkin mesh.						
$E^{N,M}$	–	8.3319e-02	1.2245e-01	1.9830e-01	2.5413e-01	1.9134e-01
$\wedge^{N,M}$	–	−5.5548e-01	−6.9549e-01	−3.5788e-01	4.0943e-01

**Table 2:** Comparison of $E_{\varepsilon }^{N,M},E^{N,M},\wedge_{\varepsilon }^{N,M}$, and $\wedge^{N,M}$ for Example 1.8 of the proposed scheme with results in [17].

ε↓N→	16	32	64	128	256	512
M→	10	20	40	80	160	320
Present method						
$2^{-8}$	3.4080e-03	2.4496e-03	1.4330e-03	7.6951e-04	3.9831e-04	2.0904e-04
	4.7638e-01	7.7351e-01	8.9703e-01	9.5005e-01	9.3011e-01	
$2^{-10}$	3.4872e-03	2.4921e-03	1.4856e-03	7.9557e-04	4.1136e-04	2.0989e-04
	4.8471e-01	7.4632e-01	9.0098e-01	9.5159e-01	9.7077e-01	
$2^{-12}$	3.5570e-03	2.5397e-03	1.5051e-03	8.0549e-04	4.1606e-04	2.1136e-04
	4.8600e-01	7.5480e-01	9.0192e-01	9.5308e-01	9.7709e-01	
$2^{-14}$	3.5907e-03	2.5561e-03	1.5114e-03	8.0836e-04	4.1747e-04	2.1212e-04
	4.9032e-01	7.5806e-01	9.0282e-01	9.5333e-01	9.7679e-01	
$2^{-16}$	3.6049e-03	2.5624e-03	1.5127e-03	8.0924e-04	4.1789e-04	2.1235e-04
	4.9246e-01	7.6037e-01	9.0249e-01	9.5344e-01	9.7668e-01	
$2^{-18}$	3.6098e-03	2.5648e-03	1.5133e-03	8.0950e-04	4.1802e-04	2.1242e-04
	4.9307e-01	1.5133e-03	9.0260e-01	9.5346e-01	9.7665e-01	
$2^{-20}$	3.6119e-03	2.5656e-03	1.5136e-03	8.0960e-04	4.1806e-04	2.1244e-04
	4.9346e-01	7.6131e-01	9.0270e-01	9.5350e-01	9.7679e-01	
$2^{-22}$	3.6126e-03	2.5660e-03	1.5138e-03	8.0963e-04	4.1808e-04	2.1245e-04
	4.9352e-01	7.6135e-01	9.0284e-01	9.5348e-01	9.7666e-01	
$2^{-24}$	3.6128e-03	2.5660e-03	1.5138e-03	8.0963e-04	4.1808e-04	2.1245e-04
	4.9360e-01	7.6135e-01	9.0284e-01	9.5348e-01	9.7666e-01	
$E^{N,M}$	3.6128e-03	2.5660e-03	1.5138e-03	8.0963e-04	4.1808e-04	2.1245e-04
$\wedge^{N,M}$	4.9360e-01	7.6135e-01	9.0284e-01	9.5348e-01	9.7666e-01	
Results in [17] using the adaptive grid method.						
$E^{N,M}$	–	2.8299e-01	1.7036e-01	1.1805e-01	7.4251e-02	4.1879e-02
$\wedge^{N,M}$	–	7.322e-01	5.292e-01	6.689e-01	8.262e-01	
Results in [17] using the Shishkin mesh.						
$E^{N,M}$	–	9.9532e-02	5.4894e-02	3.5442e-01	2.9823e-01	2.1963e-01
$\wedge^{N,M}$	–	−8.585e-01	−2.6907	2.490e-01	4.388e-01

**Table 3:** $E_{\varepsilon }^{N,M},E^{N,M}$, $\wedge_{\varepsilon }^{N,M}$, and $\wedge^{N,M}$ for Example 1.9 of the proposed scheme with results in [13].

ε↓N→	16	32	64	128	256
M→	10	20	40	80	160
$2^{-8}$	2.3456e-02	1.4948e-02	8.5282e-03	4.5333e-03	2.3339e-03
	6.5000e-01	8.0964e-01	9.1168e-01	9.5782e-01	
$2^{-10}$	2.3615e-02	1.5196e-02	8.6211e-03	4.5716e-03	2.3516e-03
	6.3601e-01	8.1775e-01	9.1517e-01	9.5906e-01	
$2^{-12}$	2.3396e-02	1.5306e-02	8.6161e-03	4.5788e-03	2.3562e-03
	6.1216e-01	8.2899e-01	9.1207e-01	9.5851e-01	
$2^{-14}$	2.3345e-02	1.5292e-02	8.6056e-03	4.5824e-03	2.3570e-03
	6.1034e-01	8.2943e-01	9.0917e-01	9.5915e-01	
$2^{-16}$	2.3337e-02	1.5285e-02	8.6054e-03	4.5839e-03	2.3572e-03
	6.1050e-01	8.2880e-01	9.0867e-01	9.5950e-01	
$2^{-18}$	2.3335e-02	1.5284e-02	8.6065e-03	4.5834e-03	2.3573e-03
	6.1047e-01	8.2852e-01	9.0901e-01	9.5928e-01	
$2^{-20}$	2.3335e-02	1.5284e-02	8.6074e-03	4.5837e-03	2.3572e-03
	6.1047e-01	8.2837e-01	9.0906e-01	9.5944e-01	
$2^{-22}$	2.3335e-02	1.5284e-02	8.6077e-03	4.5838e-03	2.3572e-03
	6.1047e-01	8.2831e-011	9.0910e-01	9.5944e-01	
$2^{-24}$	2.3335e-02	1.5284e-02	8.6078e-03	4.5838e-03	2.3572e-03
	6.1047e-01	8.2831e-01	9.0910e-01	9.5944e-01	
$E^{N,M}$	2.3615e-02	1.5306e-02	8.6211e-03	4.5839e-03	2.3573e-03
$\wedge^{N,M}$	6.2561e-01	8.2815e-01	9.1130e-01	9.5944e-01	
Results in [13]					
$E^{N,M}$	4.1129e-2	2.2723e-2	1.2008e-2	6.179e-3	3.136e-3
$\wedge^{N,M}$	8.560e-01	9.202e-01	9.586e-01	9.784e-01

### Conclusions

Herein, the non-linear singularly perturbed Burgers’- Huxley problem was studied via a hybrid computational scheme. The numerical scheme is based on discretizing the differential equation using the implicit Euler method for the time variable and the hybrid numerical scheme composition of the midpoint Euler method in the outer layer and the cubic spline in compression method in the inner layer on a piecewise uniform Shishkin mesh for the space variable. The scheme is analyzed for ε− uniform convergence. Several examples are discussed and compared to some existing schemes in the literature to prove the effectiveness of the presented scheme. In each example, we calculated the $E_{\varepsilon }^{N,M}$, $E^{N,M}$ and the corresponding $\wedge_{\varepsilon }^{N,M}$ and $\wedge^{N,M}$ for different values of N and M. From the results in Table 1, Table 2, Table 3, we observe that the maximum point-wise error decreases as N and M increase for each value of ε goes small. We observe that the maximum point-wise errors are stable as ε goes small for each N and M and which depicts that the method is convergence with independent of ε. Our findings have confirmed that the proposed method reveals high accuracy and can yield results that are on par with, or even superior to, some available numerical schemes for tackling the SPBHEs.This achievement emphasizes the potential of the proposed scheme as a powerful tool for addressing not only the SPBHEs but also other important nonlinear partial differential equations that come across in numerous engineering and scientific contexts.

## CRediT authorship contribution statement

**Imiru Takele Daba:** Conceptualization, Methodology, Software, Visualization, Investigation, Writing – review & editing. **Genanew Gofe Gonfa:** Supervision, Data curation, Writing – original draft.

## Declaration of competing interest

The authors declare that they have no known competing financial interests or personal relationships that could have appeared to influence the work reported in this paper.

## Data Availability

No data was used for the research described in the article.
